# Hemostatic Use of Gelatine

**Published:** 1901-05-15

**Authors:** 


### HEMOSTATIC USE OF GELATINE.

If but a small proportion of the favorable results claimed for gelatine in the arrest of hemorrhage shall be attained, these few will be sufficient reward for its administration in those cases of hemorrhage which have resisted other more certain methods. Dr. J. B. Nichols (*Med. News*) has collected some of the results from the use of gelatine in the hands of various observers, and also reports a severe case of hemophilia which yielded to the treatment. Attention is called to the danger attending subcutaneous use of the drug, while intravenous injection would probably be too dangerous to be admissible. Ingestion by the mouth is excluded, since the gelatine would be altered and its coagulant properties destroyed by the process of digestion. However, a case of obstinate bleeding from a gastric ulcer is reported in which the hemorrhage was immediately arrested by the ingestion of 200 cc of a 10 percent solution administered by the mouth three times daily. The recognized dose, subcutaneously, is from 30 to 100 cc of a 2 percent sterilized aqueous solution of gelatine with 1 percent of sodium chlorid. The solution may be sterilized by keeping at a temperature of 100 degrees C. fifteen minutes daily for two or three days. Favorable results have been reported in the treatment of aortic aneurisms, hemoptysis of tuberculosis, epistaxis, uterine hemorrhages, intestinal hemorrhage in typhoid fever, hemorrhoidal bleeding, purpura hemorrhagica, and locally in 5 to 10 percent solution with .6 to 1 percent of calcium chlorid, for the arrest of hemorrhage in wounds. Of course its use is not intended to supersede established and more certain methods where they can be applied.—*Review of Medical Progress.*

